# Performance of noninvasive tools for identification of minimal liver fibrosis in patients with hepatitis B virus infection

**DOI:** 10.1002/jcla.23960

**Published:** 2021-08-17

**Authors:** Hong Zhang, XinXing Shi, Lin Wang, Yilan Zeng, Xintong Kang, Liang Huang

**Affiliations:** ^1^ Public Health Clinical Center of Chengdu Chengdu China

**Keywords:** hepatitis, liver fibrosis, Globulin–platelet ratio

## Abstract

**Background:**

Various noninvasive liver fibrosis assessment tools are available. Here, we evaluated the performance of the asparagine aminotransferase‐to‐platelet ratio index (APRI), the fibrosis‐4 index (FIB‐4), transient elastography (TE), and the globulin–platelet (GP) ratio for identifying liver fibrosis in patients with hepatitis B virus (HBV) infection.

**Methods:**

A total of 146 patients were assessed using TE, FIB‐4, APRI, the GP ratio, and liver biopsy. Three patient grouping methods were applied: any fibrosis (AF; F0 vs. F1/2/3/4); moderate fibrosis (MF; F0/1 vs. F2/3/4); and severe fibrosis (SF; F0/1/2 vs. F3/4). Receiver operating characteristic (ROC) curve analysis, univariate analyses, and multivariate logistic regression were conducted.

**Results:**

Regardless of patient‐grouping method, the area under the curve (AUC) of TE and the GP ratio were similar. Using the AF grouping method, the GP ratio showed superior performance compared with APRI and FIB‐4: the AUCs for the GP ratio, TE, APRI, and FIB‐4 were 0.76, 0.75, 0.70, and 0.66, respectively. Using the MF grouping method, the GP ratio also showed superior performance compared with APRI and FIB‐4: the AUCs for the GP ratio, TE, APRI, and FIB‐4 were 0.66, 0.68, 0.57, and 0.53, respectively. Using the SF grouping method, the AUCs for the GP ratio, TE, APRI, and FIB‐4 were not significantly different.

**Conclusion:**

Compared with FIB‐4 and APRI, the GP ratio had higher accuracy for identifying liver fibrosis, especially early‐stage fibrosis, in patients with HBV infection.

## Graphical abstract

ROC curves of noninvasive diagnostic methods for liver fibrosis (F0 vs. F1/2/3/4).

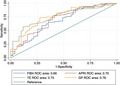

## 1 INTRODUCTION

Hepatitis B virus (HBV) infection imposes a large disease burden in China. The prevalence of HBV infection is as high as 8% in rural areas.^1^ Liver fibrosis is an underlying effect of chronic HBV infection and is related to the development of hepatocellular carcinoma.^2^ Liver fibrosis assessment is critical in evaluating the severity of HBV infections,^3^, ^4^ providing key information for patient management. Liver biopsy is the gold standard for diagnosis of liver fibrosis. However, biopsy is invasive, costly, associated with risk of sampling error,^5^ and should be performed in inpatient departments within the days of hospitalization. Despite its good safety record^6^, ^7^ and high accuracy, patient acceptance rates are low. An ideal method to assess liver fibrosis should be rapid, safe, economical, accessible, and accurate.

Simple algorithms for assessment of serum biomarkers of liver fibrosis have been developed. The American Association for the Study of Liver Diseases recommended the age–aspartate aminotransferase (AST)–platelet (PLT)–alanine aminotransferase (ALT) index (FIB‐4)^8^ and the AST‐to‐PLT ratio index (APRI) as noninvasive tools for liver fibrosis assessment.^3^ Compared with liver biopsy, noninvasive liver fibrosis assessment tools are more widely accepted by patients are used by many clinicians.^9^, ^10^ Transient elastography (FibroScan, TE) is another convenient way to assess liver fibrosis, reducing the need for liver biopsy.^11^ TE showed good predictive performance for HBV‐infected patients in a previous study.^12^ However, this technique requires special equipment and is not accessible in all settings. The globulin (GLB)–PLT (GP) ratio was first proposed by Liu^13^ and subsequently independently verified. The GP ratio was used to evaluate patients with high HBV‐DNA loads and mildly elevated ALT levels. The authors concluded that GP was a more accurate tool than APRI and FIB‐4 for diagnosis of cirrhosis in CHB patients with high HBV‐DNA loads and mildly elevated ALT levels.^14^

Although noninvasive tools show good performance in the diagnosis of the later stages of liver fibrosis, they may have not been validated for earlier stages.^15^ In this study, we evaluated noninvasive methods for identification of patients with at least minimal liver fibrosis (F1). We applied three patient grouping strategies including no liver fibrosis (F0) versus any degree of liver fibrosis (F1/2/3/4). We collected data on 146 patients with HBV infection, who have undergone liver biopsies, TE, blood tests, and liver function tests. We assessed the relative performance of the GP ratio, FIB‐4, APRI, and TE using liver biopsy as the gold standard. We used multivariate logistic regression to identify prognostic factors for liver fibrosis.

## 2 METHODS

### 2.1 Patients

This study included 146 patients with HBV infection. All patients were Chinese. The study was approved by the Ethics Committee of Chengdu Public Health Clinical Center. Written informed consent was obtained from participants prior to liver biopsy and blood tests. The study was complied with the ethical guidelines set out in the 2008 Declaration of Helsinki. Patients with liver inflammations attributed to factors other than HBV infection (e.g., alcohol abuse, hepatitis C virus infection, autoimmune hepatitis, or drug‐induced hepatitis) were excluded.

### 2.2 Pathology

Samples were reviewed by two pathologists. Stages of fibrosis were determined according to the METAVIR^16^ system as follows: no or mild fibrosis, no fibrosis or portal fibrosis without septa, F0/F1; moderate fibrosis, portal fibrosis, and few septa, F2; severe fibrosis and numerous septa without cirrhosis, F3; and cirrhosis, F4.^17^ The agreed upon diagnosis by the two pathologists was considered final. In case of disagreement, a third pathologist reviewed the case to achieve resolution.

### 2.3 Transient elastography

TE measurements were performed on the right lobe of the liver to obtain liver stiffness measure values. The results were expressed in kilopascals (kPa). The median value of 10 successful measurements was considered representative of liver stiffness. The duration of examination was <5 min. TEs were carried out within 1 week of liver biopsy.

### 2.4 Laboratory tests

Liver function tests and routine blood tests were carried out within 1 week of liver biopsy. The FIB‐4 score^8^ was calculated as [age (years) × AST (U/L)]/[PLT (10^9^/L) × ALT(U/L)^−2^]. The APRI score was calculated as [AST (U/L) / AST upper normal limit]/PLT (10^9^/L). Upper limits of 37 U/L were used for ALT and AST in both women and men by local convention.

### 2.5 Patient grouping

Three strategies were used for patients grouping: any fibrosis (AF; F0 vs. F1/2/3/4); moderate fibrosis (MF; F0/1 vs. F2/3/4); and severe fibrosis (SF; F0/1/2 vs. F3/4). The AF grouping method was used to differentiate patients with or without at least minimal liver fibrosis. The MF grouping method was used to differentiate patients with or without progressive liver fibrosis. The SF grouping method was used to differentiate patients with or without significant fibrosis or liver cirrhosis.

### 2.6 Statistical analysis

Statistical analysis was performed using STATA/SE 14.1 software (StataCorp). Normally distributed continuous data were presented as means and standard deviations (SDs), while nonnormally distributed continuous data were presented as medians and ranges. Comparisons between two groups were performed using Student's *t* tests or Wilcoxon rank sum tests. Fisher's exact tests were used to assess differences in count data. The area under the curves (AUC) was calculated using receiver operator characteristic (ROC) curve analysis. Multivariable logistic regression with stepwise variable selection was applied to fit the data analyzed using different grouping methods. Values of *p* < 0.05 were considered statistically significant.

## 3 RESULTS

### 3.1 General characteristics of the patients

Among the 146 patients with HBV infection, 102 (69.86%) were male. The average age was 39.7 years (SD 9.43 years). The average body mass index was 23.2 (SD 3.02). Among the 146 patients, 51 (34.9%) cases were staged as F0, 54 (37.0%) as F1, 27 (18.5%) as F2, 10 (6.9%) as F3, and 4 (2.7%) as F4.

The median ALT level was 35.5 U/L (range 9–715.4 U/L), the median AST level was 30.25 U/L (range 15.5–448 U/L), and the median total bilirubin level was 12.2 μmol/L (range 0.89–78.8 μmol/L). The average PLT count was 165.9 × 10^9^/L (SD 55.5 × 10^9^/L) and the median white blood cell (WBC) count was 5.1 × 10^9^/L (range 1.7–10.5 × 10^9^/L).

### 3.2 Comparisons between grouping strategies for liver fibrosis

Using the AF grouping method, 51 patients were at stage F0 while 95 patients were at other stages. The median WBC count in F0 group was higher than in the liver fibrosis group (F1/2/3/4). The median GLB level, and gamma‐glutamyl transpeptidase level in the F0 group were lower than in the liver fibrosis group (F1/2/3/4). The mean PLT count for the F0 group was higher than in the liver fibrosis group (F1/2/3/4). These differences were statistically significant (Table 1).

**TABLE 1:** Univariate analysis of factors associated with liver fibrosis using the AF grouping method

Variables	F0 (*n* = 51)	F1/2/3/4 (*n* = 95)	*p*‐values
Age (years)^a^	37.7	40.8	0.06
ALT (U/L)^b^	37.8	35.0	0.85
AST (U/L)^a^	29.0	31.8	0.08
BMI^a^	23.3	23.1	0.69
TBIL (μmol/L)^b^	12.4	12.1	0.72
WBC (10^9^/L)^b^	5.3	4.76	0.02
PLT (10^9^/L)^a^	193.6	150.7	0.00
ALP (U/L)^b^	67.0	72.0	0.12
GGT (U/L)	21.0	25.0	0.03
ALB (g/L)^b^	44.7	44.4	0.60
GLB (g/L)^b^	28.8	30.4	0.01
Male^c^	38.0	62.0	0.35
INR^a^	1.0	1.0	0.18

Abbreviations: AF, any fibrosis (F0 vs. F1/2/3/4); ALB, albumin; ALP, alkaline phosphase; ALT, alanine aminotransferase; AST, asparagine aminotransferase; BMI, body mass index; GGT, gamma‐glutamyl transpeptidase; GLB, globulin; INR, international normalized ratio; PLT, platelet count; TBIL, total bilirubin; WBC, white blood cell count.

^a^ Data represent means; Student's *t* tests used for comparisons.

^b^ Data represent medians; Wilcoxon rank sum tests used for comparisons.

^c^ Data represent counts; Fisher's exact test used for comparisons.

Using the MF grouping method, 105 patients were at stage F0/1. The median GLB level in the F0/1 group was lower than that of the F2/3/4 group. The mean PLT count was higher in the F0/1 group than in the F2/3/4 group. These differences were statistically significant (Table 2).

**TABLE 2:** Univariate analysis of factors associated with liver fibrosis using the MF grouping method

Variables	F0/1 (*n* = 105)	F2/3/4 (*n* = 41)	*p*‐values
Age (years)^a^	39.5	40.1	0.72
ALT (U/L)^b^	38.0	31.8	0.23
AST (U/L)^a^	30.9	29.0	0.60
BMI^a^	23.0	23.5	0.37
TBIL (μmol/L)^b^	12.6	11.7	0.09
WBC (10^9^/L)^b^	5.2	4.6	0.09
PLT (10^9^/L)^a^	173.0	147.4	0.01
ALP (U/L)^b^	71.0	70.5	0.91
GGT (U/L)^b^	22.5	25.5	0.14
ALB (g/L)^b^	44.5	44.6	0.44
GLB (g/L)^b^	29.4	30.3	0.02
Male^c^	70.0	30.0	0.42
INR^a^	1.0	1.0	0.39

Abbreviations: ALB, albumin; ALP, alkaline phosphase; ALT, alanine aminotransferase; AST, asparagine aminotransferase; BMI, body mass index; GGT, gamma‐glutamyl transpeptidase; GLB, globulin; INR, international normalized ratio; MF, moderate fibrosis (F0/1 vs. F2/3/4); PLT, platelet count; TBIL, total bilirubin; WBC, white blood cell count.

^a^ Data represent means; Student's *t* tests used for comparisons.

^b^ Data represent medians; Wilcoxon rank sum tests used for comparisons.

^c^ Data represent counts; Fisher's exact test used for comparisons.

Using the SF grouping method, only 14 patients were classified as stage F3/4. The median GLB level in these patients was higher compared with the F0/1/2 group. However, the differences between the two groups were not statistically significant (Table 3).

**TABLE 3:** Univariate analysis of factors associated with liver fibrosis using the SF grouping method

Variables	F0/1/2 (*n* = 132)	F3/4 (*n* = 14)	*p*‐values
Age (years)^a^	39.5	41.3	0.51
ALT (U/L)^b^	35.3	34.0	0.75
AST (U/L)^a^	30.0	35.0	0.18
BMI^a^	23.1	23.0	0.66
TBIL (μmol/L)^b^	12.0	13.0	0.19
WBC (10^9^/L)^b^	5.1	4.0	0.15
PLT (10^9^/L)^a^	169.0	134.2	0.03
ALP (U/L)^b^	71.0	75.0	0.41
GGT (U/L)^b^	23.0	33.0	0.09
ALB (g/L)^b^	44.5	44.5	0.53
GLB (g/L)^b^	29.5	31.7	0.07
Male^c^	91.0	9.0	0.60
INR^a^	1.0	1.1	0.11

Abbreviations: ALB, albumin; ALP, alkaline phosphase; ALT, alanine aminotransferase; AST, asparagine aminotransferase; BMI, body mass index; GGT, gamma glutamyl transpeptidase; GLB, globulin; INR, international normalized ratio; PLT, platelet count; SF, severe fibrosis (F0/1/2 vs. F3/4); TBIL, total bilirubin; WBC, white blood cell count.

^a^ Data represent means; Student's *t* tests used for comparisons.

^b^ Data represent medians; Wilcoxon rank sum tests used for comparisons.

^c^ Data represent counts; Fisher's exact test used for comparisons.

We next stratified patients into two groups to validate the performance of different noninvasive tools in the following analysis. However, the characteristics of the two groups may not be a biological representative of liver fibrosis progression. The analysis of patient characteristics of each group (F0/1/2/3/4) is presented in supplementary data (Table S1).

### 3.3 Multivariable analysis

Multivariable logistic regression with stepwise variable selection was used for identifying relevant variables predicting liver fibrosis levels. Three models were constructed according to the three grouping methods. The results indicated that PLT count and GLB level were statistically significant predictors of fibrosis using the AF and MF grouping methods. PLT count, ALT level, and GLB level remained after stepwise variable selection using the SF grouping method. However, only GLB was statistically significant (Table 4). Generally, we found that lower PLT counts and higher GLB levels correlated with higher risks of liver fibrosis and vice versa.^18^ The results of multivariable analysis justified further study of the GP ratio as a predictor of fibrosis.

**TABLE 4:** Logistic regression analysis of factors associated with liver fibrosis using different grouping methods

Grouping methods	Variables	OR	95% CI		*p*‐values
AF^a^	GLB	1.14	1.02	1.28	0.02
	PLT	0.98	0.98	0.99	0.00
MF^b^	PLT	0.99	0.99	1.00	0.03
	GLB	1.14	1.02	1.26	0.02
SF^c^	PLT	0.99	0.98	1.00	0.09
	ALT	1.01	1.00	1.02	0.06
	GLB	1.21	1.03	1.42	0.02

Abbreviations: ALT, alanine aminotransferase; CI, confidence interval; GLB, globulin; PLT, platelet.

^a^ AF, any fibrosis (F0 vs. F1/2/3/4).

^b^ MF, moderate fibrosis (F0/1 vs. F2/3/4).

^c^ SF, severe fibrosis (F0/1/2 vs. F3/4).

### 3.4 Globulin–Platelet ratio

Based on the results of multivariable analysis, GLB levels and PLT counts were statistically significantly associated with different liver fibrosis stages. The GP ratio could be a predictor of liver fibrosis. The GP ratio was calculated as GLB (g/L)/PLT (10^9^/L) × 10.

### 3.5 Area under the curve comparisons

**FIGURE 1:**
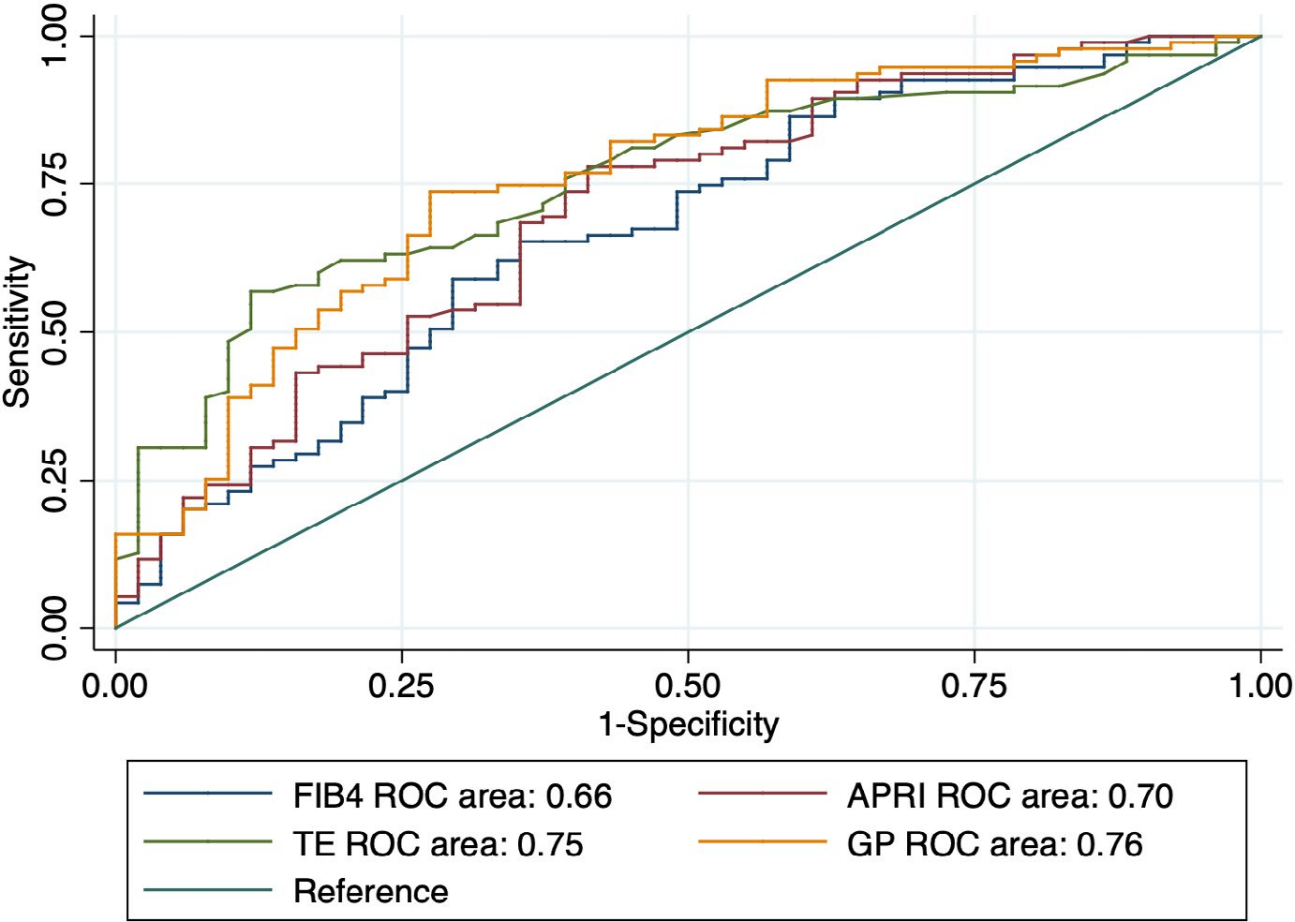
ROC curves of noninvasive diagnostic methods for liver fibrosis using the AF grouping method. AF, any fibrosis (F0 vs. F1/2/3/4)

**FIGURE 2:**
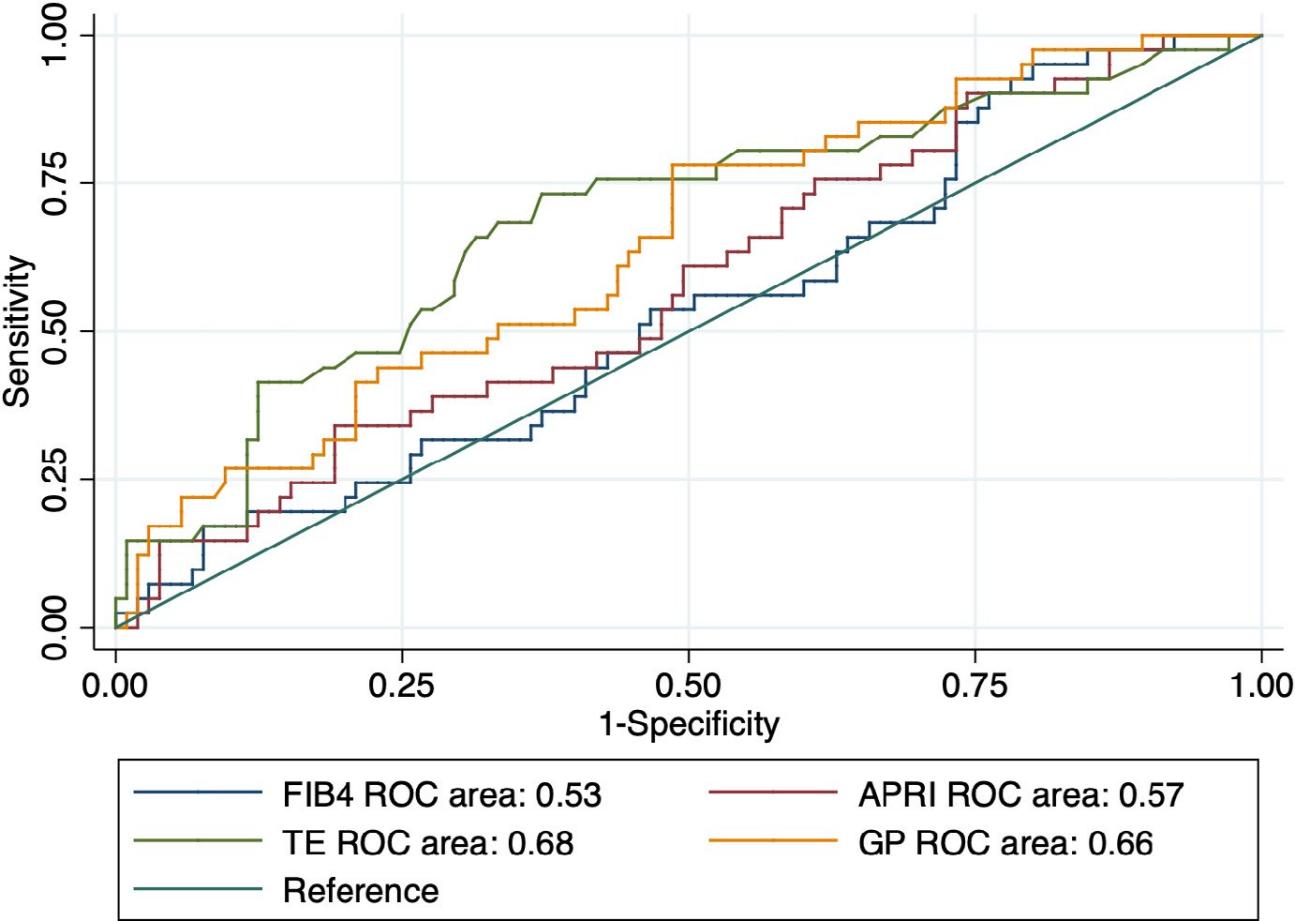
ROC curves of noninvasive diagnostic methods for liver fibrosis using the MF grouping method. MF, moderate fibrosis (F0/1 vs. F2/3/4)

**FIGURE 3:**
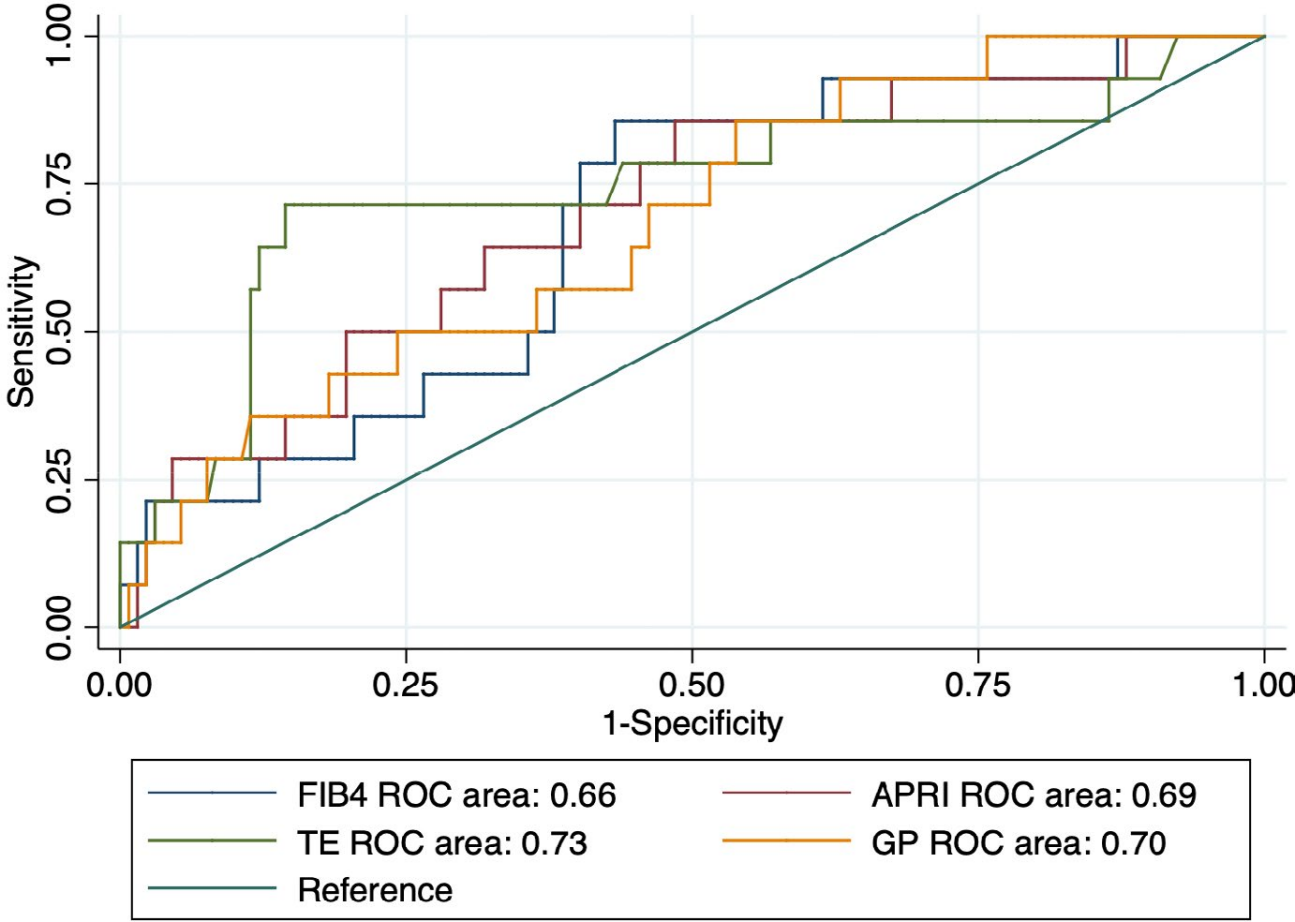
ROC curves of noninvasive diagnostic methods for liver fibrosis using the SF grouping method. SF, severe fibrosis (F0/1/2 vs. F3/4)

ROC curve analysis was used to compare the performance of different noninvasive methods for assessing liver fibrosis. We evaluated TE, FIB‐4, APRI, and the GP ratio (Figures 1, 2, 3). For all grouping methods, the AUCs of TE and the GP ratio were similar. Using the AF grouping method, the GP ratio showed superior performance to APRI and FIB‐4. The AUCs for the GP ratio, TE, APRI, and FIB‐4 were 0.76, 0.75, 0.70, and 0.66, respectively. Using the MF grouping method, the GP ratio showed a higher AUC than APRI and FIB‐4. The AUCs for GP ratio, TE, APRI, and FIB‐4 were 0.66, 0.68, 0.57, and 0.53, respectively. Using the SF grouping method, the differences in AUCs between the GP ratio and TE, APRI, and FIB‐4 were not statistically significant (Table 5). Thus, the GP ratio had better performance compared with FIB‐4 and APRI using the AF and MF grouping methods.

**TABLE 5:** Comparison of AUCs for the GP ratio, APRI, FIB‐4, and TE

	AF^a^		MF^a^		SF^a^	
	AUC (95%CI)	*p*‐values^b^	AUC (95%CI)	*p*‐values^b^	AUC (95%CI)	*p*‐values^b^
APRI	0.70 (0.61–0.79)	0.03	0.57 (0.47–0.68)	0.02	0.69 (0.54–0.84)	0.85
TE	0.75 (0.67–0.83)	0.94	0.68 (0.58–0.78)	0.56	0.73 (0.55–0.98)	0.69
FIB−4	0.66 (0.57–0.76)	0.03	0.53 (0.43–0.63)	0.03	0.66 (0.52–0.80)	0.61
GP ratio	0.76 (0.67–0.84)	/	0.66 (0.56–0.75)	/	0.70 (0.55–0.84)	/

Abbreviations: APRI, asparagine aminotransferase‐to‐platelet ratio index; AUC, area under the curve; FIB‐4, age–aspartate aminotransferase–platelet–alanine aminotransferase index; GP ratio, globulin–platelet ratio; TE, transient elastography.

^a^ AF, MF, and SF were different patients grouping methods for liver fibrosis stage. AF, any fibrosis (F0 vs. F1/2/3/4); MF, moderate fibrosis (F0/1 vs. F2/3/4); SF, severe fibrosis (F0/1/2 vs. F3/4).

^b^ The *p*‐values were obtained by comparisons of AUCs between GP ratio and other methods.

### 3.6 Cut‐off values for noninvasive diagnosis of liver fibrosis

We used the maximal Youden's index (sensitivity + specificity − 1) to identify optimal cut‐off values for GP, APRI, FIB‐4, and TE using the AF grouping method (F0 vs. F1/2/3/4); which were 2.12, 0.42, 1.80, and 8.20, respectively (Table 6). Using these cut‐off values, the results indicated that the GP ratio had higher sensitivity and moderate specificity compared with APRI, FIB‐4, and TE. TE showed a higher specificity but lower sensitivity. The higher sensitivity of the GP ratio would make it more suitable for patient screening.

**TABLE 6:** Optimal cut‐off values for noninvasive diagnostic methods for liver fibrosis using the AF grouping method

	Cut‐off value	Sensitivity (%)	Specificity (%)	PPV (%)^a^	NPV (%)^b^	Youden's index
GP	2.12	86	61	87	47	0.43
APRI	0.42	77	59	77	58	0.37
FIB‐4	1.80	65	65	78	50	0.30
TE	8.20	57	88	90	51	0.45

Abbreviations: AF, any fibrosis (F0 vs. F1/2/3/4); ^a^PPV, positive predictive value；^b^ NPV, negative predictive value.

## 4 DISCUSSION

Platelet count has been shown to be a predictor of liver fibrosis.^19^ In our study, the PLT count showed predictive value using the AF and MF grouping methods in multivariate logistic regression. However, the predictive value of PLT was not statistically significant using the SF grouping method. We considered that on the basis of this result, we could not reject the hypothesis that PLT count may have predictive value for diagnosis of liver fibrosis assessment.

The GP ratio is potentially a suitable tool for assessing liver fibrosis in patients with HBV infection, especially for those with minimal liver fibrosis. The AUCs of the GP ratio were superior to those of APRI and FIB‐4 using the AF and MF grouping methods. In contrast, the AUCs of TE and the GP ratio were similar using all grouping methods.

TE is a rapid and noninvasive technique that can easily be performed and has become more accessible in hospitals. However, the performance of TE is correlated with liver biochemistry: if liver function is not stable, this may compromise the accuracy of TE.^20^ TE accuracy can be increased if combined with other noninvasive tools including serum markers.^21^ The GP ratio had the highest sensitivity and TE had the highest specificity for diagnosis minimal liver fibrosis. The two tools combined would be expected to be highly effective for identifying patients with minimal liver fibrosis.

The APRI is frequently used for liver fibrosis assessments in patients with nonalcoholic fatty liver disease and nonalcoholic steatohepatitis.^11^, ^22^ A similar study conducted in patients with HBV infection in China documented unsatisfactory accuracies of FIB‐4 and APRI.^23^ Another study of the performance of FIB‐4 and APRI performance in patients with HBV‐associated hepatocellular carcinoma showed low diagnostic accuracies.^24^ The APRI was initially designed with various factors in mind rather than specifically for HBV infection. The distinct underlying biological processes of HBV infection may cause disparities and compromise diagnostic performance. FIB‐4 was reportedly valuable for detecting significant fibrosis and cirrhosis in HBV‐infected patients, but had suboptimal accuracy in excluding fibrosis and cirrhosis.^25^

In our study, the GP ratio showed similar performance to TE in using the AF grouping system. Compared with the APRI and FIB‐4, the GP ratio had higher sensitivity for detecting minimal liver fibrosis. These results indicated the advantages of the GP ratio over TE, given that patients with severe obesity and elevated liver stiffness have the greatest risks of discordance with liver biopsy.^26^ Although several noninvasive options are available to assess liver fibrosis including the GP ratio, they should only be used for screening patients. The accuracy of these methods is not comparable with that of magnetic resonance elastography^27^ or liver biopsy.

We followed up most patients with HBV infection in the outpatient department. Most have limited examination results compared with inpatients. The most common tests were routine blood and liver function tests and were repeatedly obtained every 1–6 months in these patients. Compared with FIB‐4 and APRI, the GP ratio was more suitable for quickly distinguishing patients at stages F0 vs. F1–4. The GP ratio can be easily calculated using data from routine blood and liver functions tests and involves a less complex mathematical calculation than FIB‐4 and APRI. The GP ratio should be used to identify patients who would require further examinations such as TE, liver biopsy, or magnetic resonance elastography.

Our results indicate that compared with the GP ratio, the FIB‐4 and APRI methods may be less suitable for patients with HBV infection. HBV is a major cause of liver injury in China. The GP ratio may be a promising tool for diagnosis of HBV‐infected patients in outpatient departments. However, larger studies for further validation are warranted.

## CONFLICT OF INTEREST

The authors declare that they have no conflict of interest.

## DATA AVAILABILITY STATEMENT

The data supporting the findings of this study are available from the corresponding author on request.

## Supporting information

Supplementary Material
