# Supplementary material for: Performance of noninvasive tools for identification of minimal liver fibrosis in patients with hepatitis B virus infection
Source: J Clin Lab Anal. 2021 Aug 17;35(9):e23960. doi: 10.1002/jcla.23960 (PMC8418491; doi:10.1002/jcla.23960)
Supplement: Supplementary file 1 [file JCLA-35-e23960-s001.docx]

Supplementary data： Analysis for patients basic characteristics for different

Variables	F0(n=51)	F1(n=54)	F2(n=27)	F3(n=10)	F4(n=4)	*P*-values
Age(years)[1]	37.67	41.11	39.56	41.80	40.00	0.56
ALT(U/L) [2]	37.80	39.55	30.70	43.35	29.35	0.25
AST(U/L)[2]	29.00	35.25	27.50	32.80	39.75	0.02
BMI[1]	23.28	22.73	23.50	23.94	22.54	0.69
TBIL(μmol/L)[2]	12.42	13.05	9.35	13.96	14.47	0.03
WBC(10^9/L)[2]	5.30	4.81	4.63	5.16	3.12	0.13
PLT(10^9/L)[1]	193.57	152.60	153.78	146.30	100.00	0.00
ALP(U/L)[2]	67.00	73.00	69.00	66.50	97.00	0.14
GGT(U/L)[2]	21.00	24.50	25.00	39.00	31.00	0.12
ALB(g/L)[2]	44.69	44.39	44.71	44.05	44.31	0.84
GLB(g/L)[2]	28.77	30.40	30.20	29.73	35.19	0.05
Male[3]	38	33	21	8	2	0.34
INR[1]	0.97	0.98	0.99	1.01	1.04	0.23

[1]Means, Oneway Anova test with Bonferroni adjustment for multiple comparsions. [2] Median, Kruskal-Wallis Test witl comparisons.

[3]Count data, Fisher's exact test
